# A Fast-Response Driving Waveform Design Based on High-Frequency Voltage for Three-Color Electrophoretic Displays

**DOI:** 10.3390/mi13010059

**Published:** 2021-12-30

**Authors:** Hu Zhang, Zichuan Yi, Liming Liu, Feng Chi, Yunfeng Hu, Sida Huang, Yu Miao, Li Wang

**Affiliations:** 1 College of Electron and Information, University of Electronic Science and Technology of China Zhongshan Institute, Zhongshan 528402, China; 202021020727@std.uestc.edu.cn (H.Z.); liulmxps@126.com (L.L.); chifeng@semi.ac.cn (F.C.); shanhuyf@163.com (Y.H.); hsida0130@163.com (S.H.); myseeking@126.com (Y.M.); 2 School of Electronic Science and Engineering (National Exemplary School of Microelectronics), University of Electronic Science and Technology of China, Chengdu 611731, China; 3 School of Information Engineering, Zhongshan Polytechnic, Zhongshan 528400, China; creekxi@163.com

**Keywords:** three-color electrophoretic displays, driving waveform, response time, reference grayscale, high-frequency voltage

## Abstract

Three-color electrophoretic displays (EPDs) have the characteristics of colorful display, reflection display, low power consumption, and flexible display. However, due to the addition of red particles, response time of three-color EPDs is increased. In this paper, we proposed a new driving waveform based on high-frequency voltage optimization and electrophoresis theory, which was used to shorten the response time. The proposed driving waveform was composed of an activation stage, a new red driving stage, and a black or white driving stage. The response time of particles was effectively reduced by removing an erasing stage. In the design process, the velocity of particles in non-polar solvents was analyzed by Newton’s second law and Stokes law. Next, an optimal duration and an optimal frequency of the activation stage were obtained to reduce ghost images and improve particle activity. Then, an optimal voltage which can effectively drive red particles was tested to reduce the response time of red particles. Experimental results showed that compared with a traditional driving waveform, the proposed driving waveform had a better performance. Response times of black particles, white particles and red particles were shortened by 40%, 47.8% and 44.9%, respectively.

## 1. Introduction

As one of the most important media for human-computer interaction, displays are playing an increasingly important role in our daily life. Electrophoretic electronic paper is the most widely used reflective display due to advantages such as low power consumption, paper-like reading experience, large viewing angle, and low mass [1,2,3,4,5,6]. However, multi-color display cannot be achieved by traditional EPDs because only black particles and white particles are encapsulated [7]. The application of this electronic paper technology is limited. In 2018, a three-color EPD technology was proposed, which could solve the problem of insufficient color in EPDs [8]. However, the three-color EPD has not been popularized, and the poor performance of driving waveform is one important reason.

The driving waveform is a voltage sequence that can control the up and down movement of particles in microcapsules [9,10,11,12,13]. The color displayed by pixels is determined by the position of particles. When driving waveforms are applied to a three-color EPD, the time for it to refresh from an original image to a target image is called the response time, which can reflect the performance of the driving waveform [14,15]. An electric field model of EPDs was proposed, which provided a theoretical basis for optimizing driving waveforms [16,17,18]. Some disadvantages of traditional EPDs have been solved by optimizing driving waveforms, such as long response time, flickers, and ghost images [19,20]. For example, a driving waveform for shortening the response time of traditional EPDs was proposed. The activation pattern of this driving waveform was changed to a high-frequency voltage [21]. Flickers can be effectively reduced by high-frequency voltage. In addition, another method to shorten the response time was proposed, but the relationship between particle activity and voltage frequency was not discussed [22]. As for three-color EPDs, red particles have larger volume and mass than black or white particles, so the response time of red particles is long, which severely limits the application scenarios of three-color EPDs [23]. Therefore, it is valuable to analyze the force on particles and propose a new driving waveform. In order to realize high-performance three-color EPDs, many driving waveforms have been proposed. A damped oscillation waveform was proposed to separate red particles and black particles to improve red saturation, but the response time of red particles was increased [24]. The reference grayscale was optimized by a driving waveform, which can reduce the response time of black particles and white particles. But the response time of red particles was not reduced [25]. Moreover, a driving waveform for shortening red particle response time was proposed. The distance between particles and electrode plate was adjusted to obtain an optimal reference grayscale [26]. These driving waveforms can only reduce the response time of specific particles and are not universal, but they provide a reference direction to design a new driving waveform with high performance.

In order to realize the fast response of three-color EPDs, a driving waveform based on high-frequency voltage was proposed in this study. Using Stokes law and Newton’s second law, the forces on particles in microcapsules were analyzed. The optimal frequency and duration of the activation stage were the designed to improve particles activity. At the same time, flickers and ghost images can be reduced by the proposed driving waveform.

## 2. Principle of Three-Color EPDs

**Figure 1:**
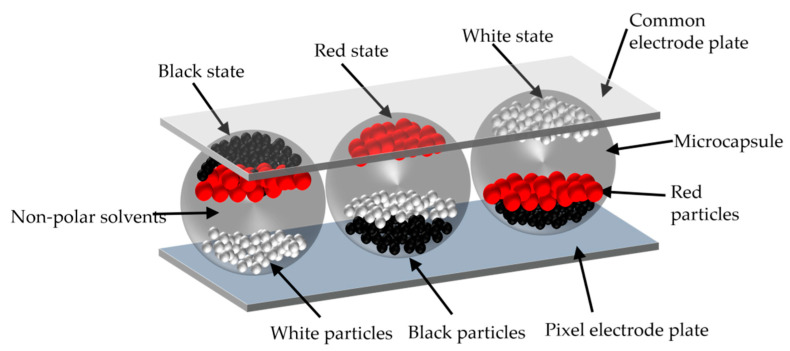
A schematic diagram of a three-color EPD. Each pixel is composed of non-polar solvents, white particles, red particles, black particles, a pixel electrode plate, and a common electrode plate. The color displayed by pixels depends on the applied voltage. The color is white when the voltage is negative polarity. The color is black when the voltage is high positive polarity. The color is red when the voltage is low positive polarity.

The structure of three pixels of three-color EPDs is shown in Figure 1. They are prepared based on microcapsule technology. Non-polar solvents, black particles, red particles, and white particles are encapsulated in microcapsules [27,28]. Microcapsules are sandwiched between a pixel electrode plate and a common electrode plate [29]. Compared with the traditional color EPD realized by a layered structure, this structure can achieve higher luminance [30,31]. Three kinds of particles in microcapsules have different sizes, polarities, and electric charges. The white particles are negatively charged, and the black and red particles are positively charged. Therefore, particles have different mobility and threshold voltages. Different colors can be displayed by applying different driving waveforms [32]. As shown on the right in Figure 1, when it is driven by a negative polarity voltage, white particles move to the top of microcapsule, and the pixel displays white. As shown on the left in Figure 1, when it is driven by a high positive polarity voltage, black particles are closer to the top of microcapsule than the red particles, so the pixel displays black. In contrast, as shown in the middle in Figure 1, when it is driven by a low positive polarity voltage, the pixel displays red. Particles remain motionless when no voltage is applied [33]. The image of three-color EPDs can be perfectly preserved for a long time.

As one of the most critical technologies of this new electrophoretic technology, the driving waveform directly affects the motion state of particles. Therefore, it is necessary to analyze the force on particles in microcapsules to optimize the driving waveform. In microcapsules, the density of particles is approximately equal to that of the non-polar solvents, which can prevent sedimentation of particles. Gravity and buoyancy of particles cancel out. Therefore, particles are affected by the combined viscous force of non-polar solvents and the electric field force. The acceleration of particles can be calculated according to Newton’s second law, as shown in Equation (1).

(1) $$F=\frac{Uq}{d}-6\pi \eta vR=ma=m\frac{dv}{dt}$$

where F is the resultant force; U is the voltage applied to applied to pixels in real time; q is the amount charge of a particle; d is the distance between the pixel electrode plate and the common electrode plate; η is the liquid viscosity coefficient; ν, R, m are the speed, sphere radius, and mass of the particle, respectively; a is the acceleration of the particle; and t is the duration of the voltage applied to two electrode plates. When the initial condition of the particle is static, the relationship between ν and t can be calculated by Equation (1), as shown in Equation (2).

(2) $$v=\frac{Uq}{6\pi d\eta R}\left(1-e^{-\frac{6\pi \eta R}{m}t}\right)$$

The relationship between the distance of particle movement and t can be obtained by integrating the velocity; as shown in Equation (3).

(3) $$s=\int_{0}^{t}vdt_{0}=\frac{Uq}{6\pi d\eta R}\left(t+\frac{m}{6\pi \eta R}e^{-\frac{6\pi \eta R}{m}t}\right)-\frac{qUm}{36\pi^{2}\eta^{2}R^{2}d}$$

where s is the distance of particle movement. It can be seen that s is proportional to t. In addition, the inertia of a particle is small and can be ignored. Therefore, when a high-frequency voltage is applied, particles are driven to oscillate in a certain range. The higher the frequency, the smaller the oscillation range of particles.

## 3. Experimental Results and Discussion

### 3.1. Experimental Platform

An optical experimental platform was developed by us to test performance of a traditional driving waveform and the proposed driving waveform. It can record red saturation and luminance value. The response time of red particles can be characterized by red saturation. The response time of black particles and white particles can be characterized by luminance. The platform consisted of a computer (H430, Lenovo, Beijing, China), a function generator (AFG3022C, Tektronix, Beaverton, OR, USA), a voltage amplifier (ATA-2022H, Agitek, Xi’an, China), and a colorimeter (Arges-45, Admesy, Ittervoort, The Netherlands). The three-color EPD was designed by us, and it was used as a test object in this experiment.

**Figure 2:**
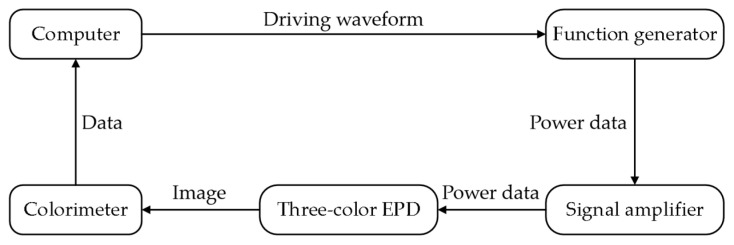
Flow chart of luminance value and red saturation data acquisition.

The workflow of the entire experiment is shown in Figure 2. First, software packages MATLAB and Arbexpress were used to edit driving waveforms and generate tfw format files, which are recognized by the function generator. Second, a universal serial bus (USB) was used to transmit the files to the function generator. The voltage amplifier was then used to amplify voltage to drive the three-color EPD. Finally, the colorimeter was placed on the three-color EPD during the experiment and Admesy software was used to collect and record luminance value and red saturation in real time.

### 3.2. Driving Waveform Design

**Figure 3:**
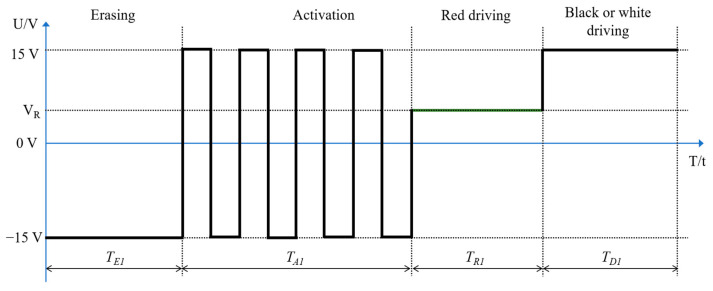
A schematic diagram of the traditional driving waveform. Its four parts are as follows: the first part is an erasing stage, the second part is an activation stage, the third part is a red driving stage, and the fourth part is a black or a white driving stage. The variable $V_{R}$ is the driving voltage of red particles, and $T_{E1}$, $T_{A1}$, $T_{R1}$, and $T_{D1}$ are the duration of the erasing stage, the activation stage, the red driving stage, and the black or white driving stage, respectively.

As shown in Figure 3, the four stages of the traditional driving waveform of three-color EPDs are as follows: an erasing stage, an activating stage, a red driving stage, and a black or white driving stage [34,35]. An original image can be erased by a −15 V voltage in the erasing stage. In the activating stage, a four-cycle square wave with a period of 340 ms and an amplitude of 30 V are used to activate particles. The activating stage and the erasing stage take a long time, which lead to a long response time of particles. A number of flickers are caused by alternating positive and negative voltages in the activation stage. Its commercial value is seriously affected due to these disadvantages. Therefore, a new driving waveform that can reduce flickers and shorten the response time is needed.

**Figure 4:**
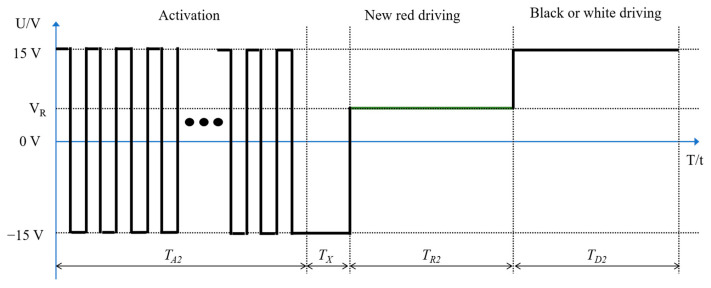
A schematic diagram of the proposed driving waveform. Its three parts are as follows: the first part is an activating stage, the second part is a new red driving stage, and the third part is a black or white driving stage. The variable $V_{R}$ is the driving voltage of red particles, and $T_{A2}$, $T_{X}$, $T_{R2}$, and $T_{D2}$ are the duration of the activation stage, the optimized reference grayscale in the red driving stage, the driving red particles in the red driving stage, and the duration of the black or white driving stage, respectively.

As shown in Figure 4, the three stages of the proposed driving waveform of three-color EPDs are as follows: an activating stage, a new red driving stage, and a black or white driving stage. Compared with Figure 3, the erasing stage is removed and the response time of particles is reduced. According to Equation (3), particles oscillate within a certain range when the voltage frequency is high. The grayscale is gradually driven to a certain value. Ghost images can be effectively reduced. After the activation stage, black particles are in the middle of microcapsules. Low red saturation is caused if red particles are driven directly. Therefore, a reference grayscale optimization voltage is added to the new red driving stage, and red particles can be driven by the positive polarity voltage in this stage. The black or white driving stage is the same as the traditional driving waveform.

### 3.3. Frequency Optimization of Activation Stage

In order to maximize particle activity after the activation stage, the optimal voltage frequency in this stage needs to be tested. When the voltage frequency is low, the alternating positive and negative voltages cause a number of flickers in the three-color EPD. The obvious flickers can be detected by the human eye when the voltage frequency is less than 50 Hz. Therefore, high-frequency voltages with frequency of 50 Hz to 150 Hz were applied to the three-color EPD. The value of $T_{A2}$ was set to 2000 ms. The activity of particles can be represented by the response time.

**Figure 5:**
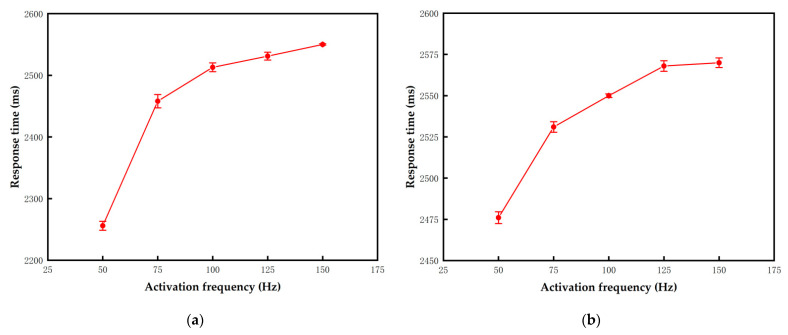
The response time of particles when they were activated by different frequency voltages. The frequency was directly proportional to the response time. The error bars were obtained by three repeated experiments. (**a**) The response time of black particles was 2256 ms when the frequency was 50 Hz. (**b**) The response time of white particles was 2476 ms when the frequency was 50 Hz.

The response time of black and white particles are shown in Figure 5a,b, respectively. The error bars were obtained by three repeated experiments. It can be proved that the voltage frequency was directly proportional to the response time. When the frequency was 50 Hz, the response time was the shortest and the activity of particles was the highest. When the frequency was too high, the duration of positive and negative voltages in a cycle was extremely short. Particles oscillated in a very small range according to Equation (3), which led to insufficient activation of particles. Therefore, the optimal frequency for the activation stage was set to 50 Hz.

### 3.4. Duration Optimization of Activation Stage

**Figure 6:**
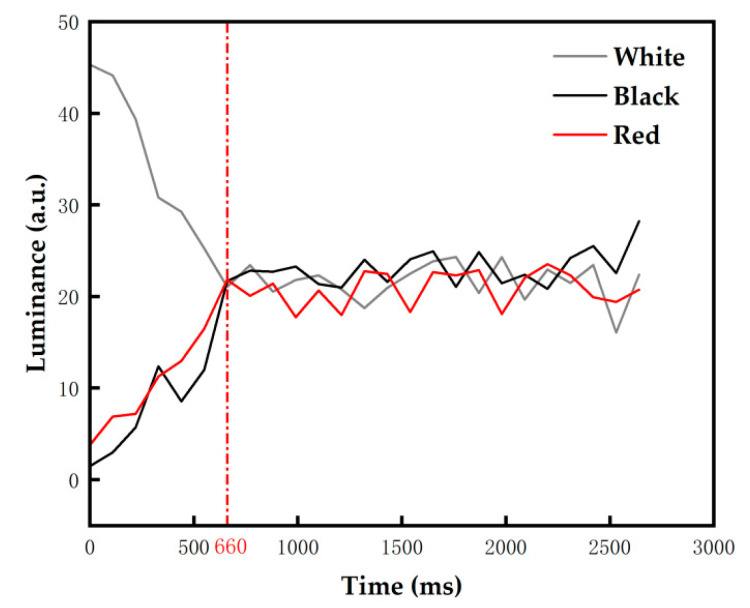
The luminance value of the three-color EPD changed with $T_{A2}$ in different initial colors. The luminance value tended to a stable value when $T_{A2}$ was longer than 660 ms.

As for the proposed driving waveform, the activation stage plays an important role in reducing ghost images because the erasing stage is removed. Hence, the optimal $T_{A2}$ needs to be determined. The frequency of this stage was set to 50 Hz, and $T_{A2}$ was set to 2000 ms to ensure that ghost images were eliminated. Initial states of the three-color EPD were set to black, red, and white. In different initial states, the luminance changed with activation time are shown in Figure 6. The experimental results showed that when $T_{A2}$ was shorter than 660 ms, the luminance of the black initial state and the red initial state could be increased, and the luminance of the white initial state could be decreased. The luminance oscillated in a small range when $T_{A2}$ was longer than 660 ms. All pixels were driven to a uniform grayscale when $T_{A2}$ was 660 ms, which proved that ghost images can be effectively reduced. Therefore, in order to reduce ghost images and minimize the response time of particles, $T_{A2}$ was set to 660 ms.

### 3.5. Driving Voltage Optimization of Red Particles

**Figure 7:**
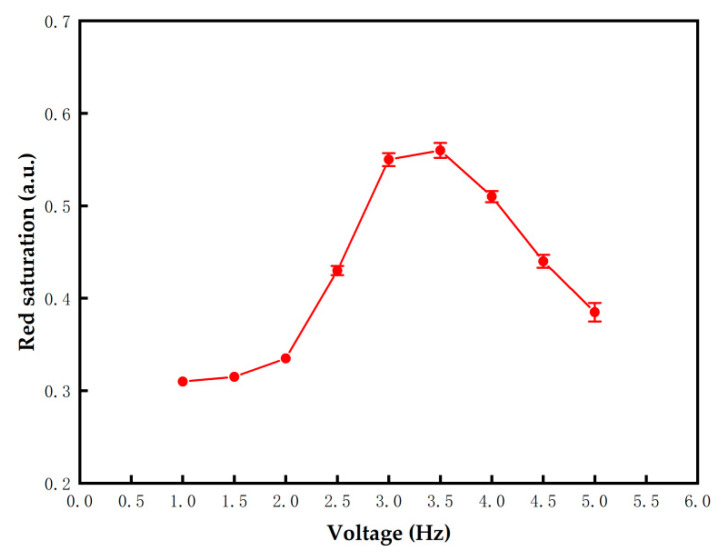
The red saturation changed with $V_{R}$ when the three-color EPD was driven by a traditional driving waveform. The error bars were obtained by three repeated experiments. Red particles cannot be driven when $V_{R}$ was too low, and black particles were also driven to the common electrode plate when $V_{R}$ was too high. Red particles can be effectively driven when the $V_{R}$ s were set to 3 V, 3.5 V, and 4 V.

The traditional driving waveform was used in this experiment. The effect of direct current (DC) voltage on red particles was tested. The value of $T_{E1}$ was set to 500 ms, and the $V_{R}$s were set to 1 V to 5 V. The red saturation changes with $V_{R}$ is shown in Figure 7. The error bars were obtained by three repeated experiments. The result showed that the red saturation was directly proportional to $V_{R}$ when $V_{R}$ was increased from 1 V to 3.5 V. This phenomenon proved that red particles cannot be driven when $V_{R}$ was lower than the threshold voltage of red particles. However, the red saturation was inversely proportional to $V_{R}$ when $V_{R}$ was increased from 3.5 V to 5 V. The voltage exceeded the threshold voltage of black particles. Therefore, black particles were also driven to the common electrode plate, which causes the red saturation to decrease. Red particles can be effectively driven when the $V_{R}$s were set to 3 V, 3.5 V, and 4 V. The red saturation reached 0.55, 0.56, and 0.51, respectively.

### 3.6. New Red Driving Stage Optimization

**Figure 8:**
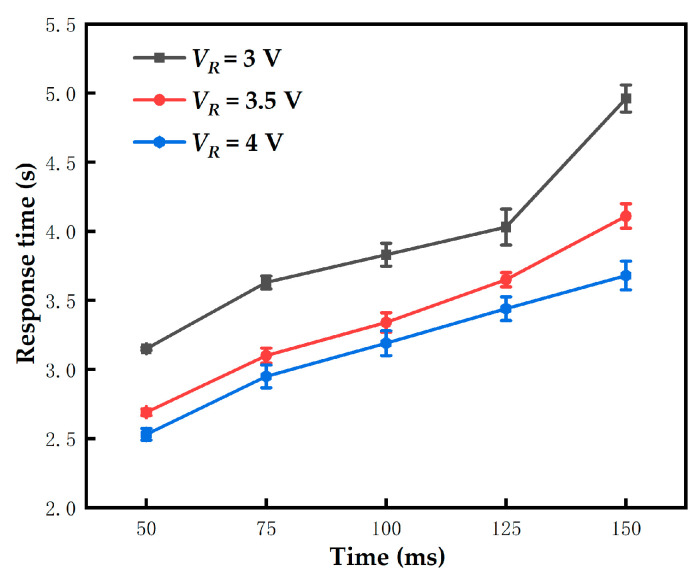
The response time of red particles changed with $V_{R}$ and $T_{X}$. The error bars were obtained by five repeated experiments. The response time was directly proportional to $T_{X}$ and inversely proportional to $V_{R}$. The optimal response time was 2.53 s when $V_{R}$ was 4 V and $T_{X}$ was 50 ms.

In order to shorten response time of red particles and improve red saturation, the new red driving stage was optimized in two aspects: $T_{X}$ and $V_{R}$. The frequency of the activation stage was set to 50 Hz; $T_{A2}$ was set to 660 ms; the $T_{X}$s were set to 0 ms to 150 ms; and the $V_{R}$s were set to 3 V, 3.5 V, and 4 V. The time when the red saturation of pixels reached 0.5 was called the response time of red particles. The influence of different $T_{X}$s and $V_{R}$s on the response time is shown in Figure 8. The error bars were obtained by five repeated experiments. The results showed that the response time of red particles was negatively correlated with $V_{R}$. According to Equation (2), the particle velocity is directly proportional to the voltage. The higher the $V_{R}$, the shorter the response time. The color displayed by pixels were a superposition of black and red when $T_{X}$ was less than 50 ms. Therefore, black particles were still close to the common electrode plate, and the red saturation cannot reach 0.5. The response time of red particles was also directly proportional to $T_{X}$. This was because as $T_{X}$ increased, the red particles moved to the pixel electrode plate. In conclusion, the optimal $T_{X}$ and $V_{R}$ were set to 50 ms and 4 V, respectively.

### 3.7. Performance of the Proposed Driving Waveform

**Figure 9:**
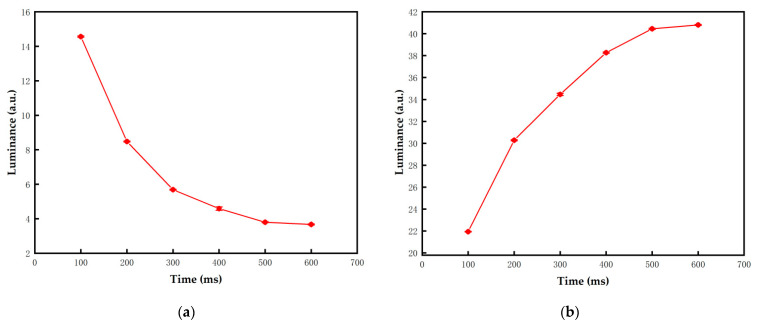
The luminance value changed with $T_{D1}$; the error bars were obtained by five repeated experiments. (**a**) The luminance value when the three-color EPD was driven to black. (**b**) The luminance value when it was driven to white.

**Figure 10:**
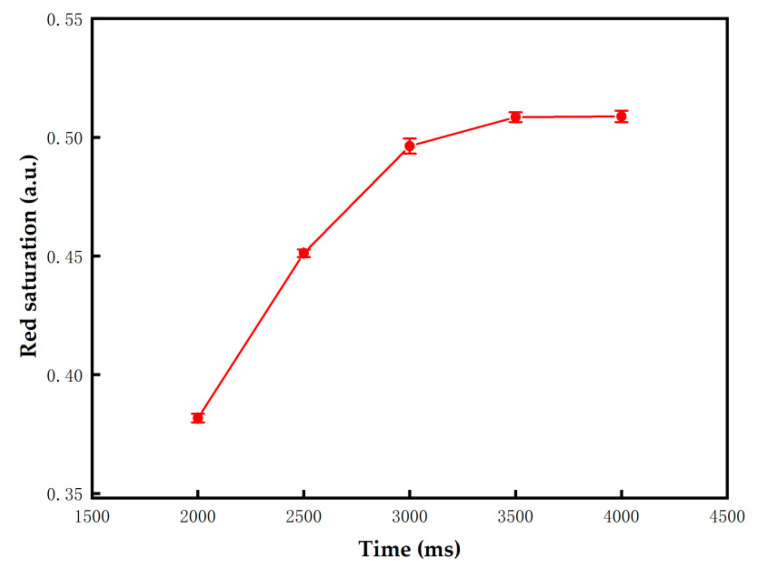
The red saturation changed with $T_{R1}$; the error bars were obtained by five repeated experiments. The red saturation was 0.5 when $T_{R1}$ was 3000 ms.

The performance of the proposed driving waveform and the traditional driving waveform was compared in a series of experiments. As for the traditional driving waveform, the parameters of each stage needed to be tested. The value of $T_{E1}$ was determined by Equation (4) when the target color was red, or it was equal to $T_{D1}$ when the target color was black or white; $V_{R}$ was set to 4 V; the $T_{R1}$s were set to 2000 ms to 4000 ms; and the $T_{D1}$s were set to 100 ms to 600 ms. As shown in Figure 9 and Figure 10, the error bars were obtained by five repeated experiments. As the driving time increased, the minimum luminance value was 3.67 when the three-color EPD was driven to black, the maximum luminance value was 40.8 when it was driven to white, and the maximum red saturation was 0.51 when it was driven to red. However, the luminance and red saturation tended to be saturated with the increase of driving time. Therefore, the optimal $T_{R1}$ and $T_{D1}$ were set to 3000 ms and 500 ms, respectively.

(4) $$15T_{E1}=V_{R}\times T_{A1}$$

**Figure 11:**
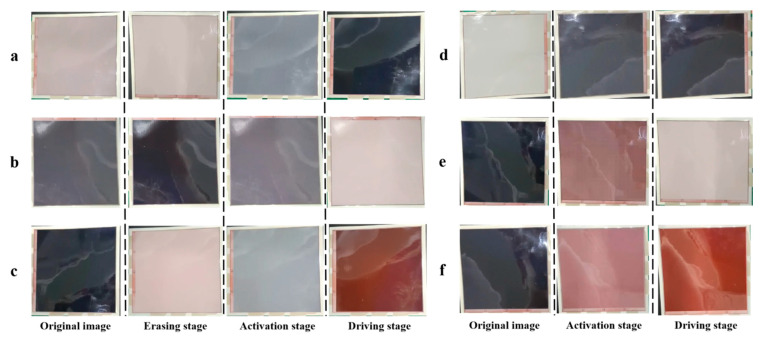
The color of each stage when the three-color EPD was driven by the two driving waveforms. (**a**) It was driven to black by the traditional driving waveform. (**b**) It was driven to white by the traditional driving waveform. (**c**) It was driven to red by the traditional driving waveform. (**d**) It was driven to black by the proposed driving waveform. (**e**) It was driven to white by the proposed driving waveform. (**f**) It was driven to red by the proposed driving waveform.

**Figure 12:**
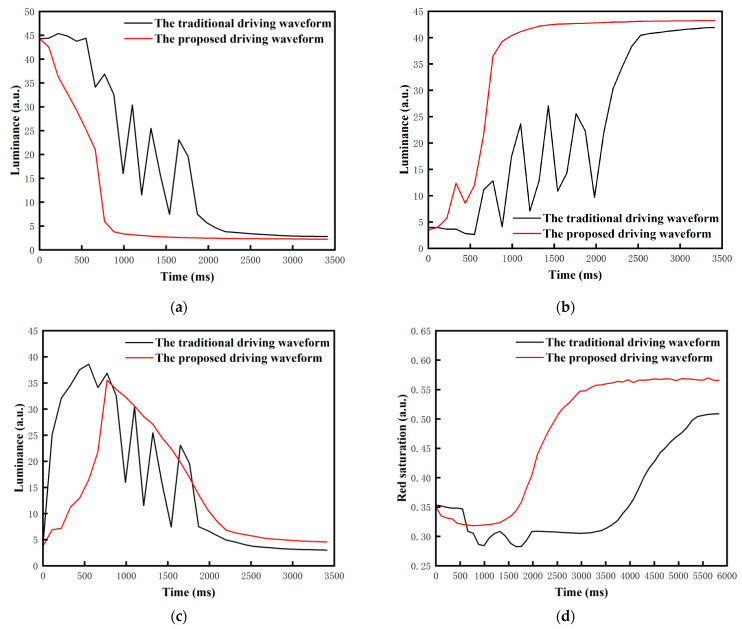
Luminance and red saturation changed with driving time when the three-color EPD was driven by the two driving waveforms. (**a**) The luminance value changed with driving time when it was driven to black. (**b**) The luminance value changed with driving time when it was driven to white. (**c**) The luminance value changed with driving time when it was driven to red. (**d**) The red saturation changed with driving time when it was driven to red.

When the three-color EPD was driven by the two driving waveforms, the color of each stage is shown in Figure 11, and flickers and the response time of particles can be seen in Figure 12. Black curves represent the change process of luminance or red saturation when the traditional driving waveform was used, and red curves represent the change process of luminance or red saturation when the proposed driving waveform was used. The results show that when it was driven by the traditional driving waveform, the response time of black particles was 2.2 s, the minimum luminance value was 3.8, and the number of flickers was 9. The response time of white particles was 2.53 s, the maximum luminance value was 40.5, and the number of flickers was 9. The response time of red particles was 5.39 s, the maximum red saturation was 0.5, and the number of flickers was 9. When the proposed driving waveform was applied, the response time of black particles was 1.32 s, the minimum luminance value was 2.85, and the number of flickers was 1. The response time of white particles was 1.32 s, the maximum luminance value was 42.2, and the number of flickers was 2. The response time of red particles was 2.97 s, the maximum red saturation was 0.55, and the number of flickers was 1. Therefore, it was proved that the response time and flickers can be effectively reduced by the proposed driving waveform. Compared with the traditional driving waveform, the response time was shortened by 40% and the number of flickers was reduced by 88.9% when the target color was black; the response time was shortened by 47.8% and the number of flickers was reduced by 77.8% when the target color was white; and the response time was shortened by 44.9% and the number of flickers was reduced by 88.9% when the target color was red.

## 4. Conclusions

In this paper, a new driving waveform based on high-frequency voltage was proposed. The response time of particles was effectively reduced because the erasing stage was removed. In addition, the activation stage was optimized to activate particles. The frequency of the activation stage was high and human eyes could not detect flickers. Therefore, flickers and ghost images were reduced. Compared with the tradition driving waveform, response time of three-color EPDs can be effectively shortened. It can provide new ideas for the research of three-color EPDs and provide users with a better visual experience.

## Data Availability

Data is contained within the article.
